# Knee joint kinematics with dynamic augmentation of primary anterior cruciate ligament repair - a biomechanical study

**DOI:** 10.1186/s40634-016-0064-2

**Published:** 2016-10-26

**Authors:** Janosch Häberli, Philipp Henle, Yves P. Acklin, Ivan Zderic, Boyko Gueorguiev

**Affiliations:** 1 Sonnenhof Orthopaedic Centre, Buchserstrasse 30, 3006 Bern, Switzerland; 2 AO Research Institute Davos, Clavadelerstrasse 8, 7270 Davos, Switzerland

**Keywords:** ACL, Knee instability, ACL repair, Dynamic Intraligamentary Stabilization

## Abstract

**Background:**

Dynamic augmentation of anterior cruciate ligament tears seems to reduce anteroposterior knee translation close to the pre-injury level. The aim of the present study is to biomechanically investigate the course of translation during a simulated early post-operative phase. It is hypothesized that anteroposterior translation is maintained at the immediate post-operative level over a simulated rehabilitation period of 50’000 gait cycles.

**Methods:**

Eight fresh-frozen human cadaveric knee joints from donors with a mean age of 35.5 (range 25–40) years were subjected to 50’000 cycles of 0°-70°-0° flexion-extension movements in a custom-made test setup. Anteroposterior translation was assessed with simulated Lachman/KT-1000 testing in 0°, 15°, 30°, 60° and 90° of flexion in knee joints treated with the novel technique initially and after 50’000 cycles testing. Statistical analysis was performed using the Wilcoxon Signed-Rank Test. The level of significance was set at *p* = 0.05.

**Results:**

Anteroposterior translation changed non-significantly for all flexion angles between cycle 0 and 50’000 (*p* = 0.39 to *p* = 0.89), except for 30° flexion, where a significant increase by 1.4 mm was found (*p* = 0.03).

**Conclusion:**

Increase in anteroposterior translation of knees treated with this dynamic augmentation procedure is low. The procedure maintains translation close to the immediate post-operative level over a simulated rehabilitation period of 50’000 gait cycles and therefore supports anterior cruciate ligament repair during biological healing.

## Background

Ruptures of the anterior cruciate ligament (ACL) are among the most common ligament injuries of the human knee - about one surgical ACL reconstruction is performed per 1000 inhabitants and year in Europe and the USA (Kohn et al. 2005). The mean age of patients suffering from an ACL lesion is between 25 and 30 years and this incident therefore has a high socioeconomic impact (Ahlden et al. 2012). The current gold standard treatment for complete ACL tears, particularly among athletes, is ligament reconstruction using an autologous or allogenic tendon graft (Vavken & Murray 2011).

The procedure was introduced by Brückner in 1966 (Brückner 1966), and achieves good results in terms of knee stability (Freedman et al. 2003; Petrigliano et al. 2006; Vavken & Murray 2011; West & Harner 2005). However, ACL reconstruction is associated with major drawbacks such as donor site morbidity in the case of an autograft tendon, a lengthy rehabilitation procedure, moderate long-term patient satisfaction, low functional scores and an increased risk for future osteoarthritis (Grindem et al. 2014; Kessler et al. 2008; Laxdal et al. 2005; Legnani et al. 2010; Meuffels et al. 2009; Pinczewski et al. 2007; Struewer et al. 2012). Laxdal et al. found that only 69.3 % of 948 patients who underwent ACL reconstruction with bone-patellar-tendon-bone (BPTB) autografts were classified as IKDC normal or nearly-normal at a median 32 month follow-up examination (Laxdal et al. 2005). The group of Pinczewski reported on 59 and 27 % kneeling pain, 10 years after bone-patellar-tendon-bone (BPTB) or hamstrings ACL reconstruction, respectively (Pinczewski et al. 2007). Meuffels et al. found no statistical difference between patients treated conservatively or operatively with respect to osteoarthritis, meniscal lesions, as well as activity level, objective and subjective functional outcome at a ten year follow-up (Meuffels et al. 2009). The group of Kessler reported on 42 % Kellgren and Lawrence grade II or higher osteoarthritis 11 years after BPTB ACL reconstruction and Streuwer et al. found 20 % grade III and IV osteoarthritis 13.5 years after BPTB ACL reconstruction (Kessler et al. 2008; Struewer et al. 2012). Grindem et al. concluded in their prospective cohort study including 100 surgically treated patients with a two year follow-up that a considerable number of patients did not fully recover after ACL injury (Grindem et al. 2014). Therefore, several attempts have been made to preserve the native ACL (Engebretsen et al. 1990; Feagin & Curl 1975; Marshall et al. 1979; Marshall et al. 1982; Murray et al. 2006; Murray et al. 2007; Silva & Sampaio 2009; Steadman et al. 2006; Steadman et al. 2012). Nowadays it is well known that isolated suturing of the ACL in most cases has shown poor clinical long-term results (Engebretsen et al. 1990; Feagin & Curl 1975; Marshall et al. 1979; Marshall et al. 1982).

More recent studies show that there is a potential for self-healing of a torn ACL if a beneficial healing environment is created (Murray et al. 2006; Murray et al. 2007; Silva & Sampaio 2009; Steadman et al. 2006; Steadman et al. 2012). The group of Steadman reported good clinical results of the “healing response” technique in skeletally immature as well as in elderly patients with a proximal ACL tear (Steadman et al. 2006; Steadman et al. 2012). In order to overcome two potential inhibitors of successful ACL healing, namely compromised blood supply and excessive motion at the scar tissue forming site, a novel treatment method called Dynamic Intraligamentary Stabilization (DIS), combining Steadman’s “healing response” technique with dynamic augmentation of a primary ACL repair, was developed and investigated (Eggli et al. 2015; Henle et al. 2015; Kohl et al. 2013; Kohl et al. 2014; Kohl et al. 2015; Kosters et al. 2015). With this technique, a polyethylene braid with a preassembled button is anchored to the femur and clamped to the DIS device, which is screwed into the tibial head.

In a static biomechanical study Schliemann et al. have shown that DIS reduced knee joint laxity close to the pre-injury level (Schliemann et al. 2015). Moreover, Kohl et al. reported that it established and maintained close contact between the two ends of the ruptured ACL (Kohl et al. 2014).

However, information about knee joint laxity with DIS over the period of rehabilitation is lacking. Therefore, the aim of this study was to biomechanically test human cadaveric knees treated with this stabilizing technique in a dynamic loading scenario. It is hypothesized that anteroposterior (AP) translation is maintained at the immediate post-operative level over a simulated rehabilitation period of 50’000 gait cycles.

## Methods

### Specimens and preparation

This study was approved by the institutional internal review board,﻿ based on the approval of the specimens' delivery by Scie﻿nce Care Ethics Committee. Eight fresh-frozen human cadaveric knees (four pairs, three male, one female, mean age 35.5 (range 25–40) years, boby mass index 17–25, no local or systemic diseases affecting joint integrity) with distal femurs, proximal tibiae and surrounding soft tissues were used. The specimens were thawed at room temperature 24 h before implantation. The proximal end of the femurs and distal end of the tibiae were embedded in polymethylmethacrylate (SCS-Beracryl, Suter-Kunststoffe AG, Fraubrunnen, Switzerland). A steel rod was secured into the tibial canal during embedding. The DIS device (Ligamys, Mathys Ltd. Bettlach, Switzerland) was implanted according to the operations manual using a small medial arthrotomy after dissection of the ACL with a scalpel at its femoral footprint. Preloading of the braid with 300 N was performed prior to its fixation to the DIS device at a pretension of 80 N in 10° flexion.

All specimens were instrumented within one working day (8 h), immediately re-frozen and then re-thawed one by one 24 h before biomechanical testing. We assumed that a mean increase of 1 mm in AP translation would be a clinically meaningful difference and that a threefold standard deviation from the mean value, namely +/- 3 mm, could be expected deviations. Based on these assumptions, a sample size of 6 specimens was necessary to reach significant differences between cycle 0 and 50’000 under a level of significance 0.05 and a power of 80 %. In order to be more conservative (power >80 %), a sample size of *n* = 8 seemed appropriate.

### Test setup

**Fig. 1:**
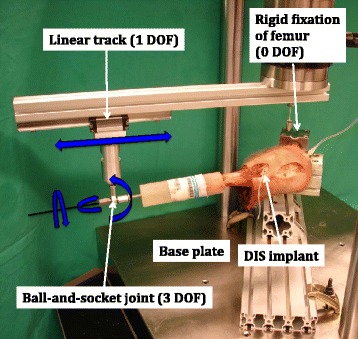
Test setup with a specimen mounted for biomechanical testing. The proximal femur is rigidly fixated to the machine base whereas the distal tibia is attached to a ball-and-socket joint that can slide freely with a linear track along the aluminum profile allowing physiological rolling and gliding of the knee. Blue arrows indicate the degrees of freedom. DOF = degrees of freedom

Each specimen was mounted on a testing machine (MTS 858 Bionix, MTS Systems Corp, Eden Prairie, MN USA) via a custom-made setup (Fig. 1). Cyclic flexion of the knee joint was achieved via rotational movement of the machine actuator. The medial epicondyle of the knee was aligned with the center of rotation of the actuator. The femoral embedding was inserted in a holder connected to the machine base. The tibiae were fixated via an intramedullary steel rod in a ball-and-socket joint to enable free rotation around the long bone axis. The ball-and-socket joint was attached to the machine actuator via an aluminum profile aligned parallel to the tibial axis. The connection between the aluminum profile and the ball-and-socket joint was achieved via a linear track allowing free linear movements along the profile for compensation of misalignment of the center of rotation. Fixation of the tibia allowed for physiological rolling and gliding mechanism in the knee joints. In order to prevent varus/valgus movements during biomechanical testing the femurs were first fixated with knees in 90° flexion and in a neutral position with no varus/valgus inclination. In this position the vertical distance between the ball-and-socket joint and the base machine plate was recorded. Then the knees were fully extended and the same distance was recorded again. Possible adjustment of the rotatory misalignment was performed by repeated rotation of the femoral embedding around the femur axis followed by measurement of the distances between the machine base and the ball-and-socket joint in both knee positions until these two respective values coincided. Possible misalignment of femoral embedding and mechanical axis of the knee in the frontal plane was adjusted by putting washers under the femoral holder. Finally, the femoral embedding was rigidly fixed in its holder. Intra-articular instillation of a test fluid was based on bovine serum (newborn calf serum, New Zealand, GIBCO Invitrogen Corporation, Lot 8097790). Sodium azide and 3 g/L EDTA were added to inhibit bacterial growth and to bind metallic ions, respectively.

### Dynamic test protocol and measurement procedure

Each specimen underwent a cyclic test simulating dynamic flexion-extension of the knee with a range of motion 0°-70°-0° (Kadaba et al. 1990) over 50’000 cycles at a frequency of 1 Hz and angular velocity of 140°/s. The test machine was controlled in angle-displacement mode so that no compensation for acceleration forces was necessary.

**Fig. 2:**
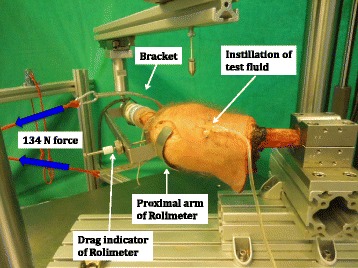
Rolimeter placed on a specimen for measurement of AP translation. Blue arrows indicate the 134 N anterior tibial force applied over a bracket at the height of the tibial tuberosity. The drag indicator of the Rolimeter is positioned on the tibial tuberosity and the proximal arm of the Rolimeter is placed on the patella. Test fluid is instilled into the supra-patellar pouch

**Fig. 3:**
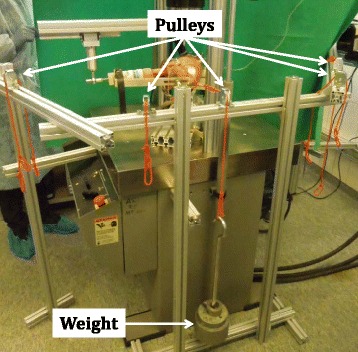
Specimen during measurement of AP translation. Five pulleys, oriented perpendicular to the 0°, 15°, 30°, 60° and 90° position of the tibia, transfer the 134 N force generated by a weight to the tibia at the height of the tuberosity

AP translation of the knee joint was assessed with a Rolimeter (Aircast, Summit, NJ, USA) in 0°, 15°, 30°, 60° and 90° flexion initially and after 50’000 cycles testing. For a standardized procedure of the measurements, the drag indicator of the Rolimeter was oriented horizontally and positioned on the tibial tuberosity, which was marked beforehand. The proximal arm of the Rolimeter was positioned on the patella, which was pushed firmly against the trochlear groove. The distal arm of the Rolimeter was attached to the tibial embedding. The Rolimeter was kept in place during all five measurements with different knee flexion angles. For each measurement, a standardized force of 134 N was applied perpendicularly to the tibial longitudinal axis at the height of the tibial tuberosity via a weight attached over a cord to a bracket (Fig. 2). Five pulleys, oriented perpendicular to the 0°, 15°, 30°, 60° and 90° position of the tibia, transmitted the 134 N force to the tibial tuberosity and simulated the situation during a clinical examination of the knee joint with a KT-1000 device (Fig. 3) (Herbort et al. 2013; Loh et al. 2003; Petersen et al. 2007; Schliemann et al. 2015).

### Data evaluation and analysis

Statistical analysis was performed using SPSS software package (version 22, IBM SPSS, Chicago, Illinois, USA). Descriptive statistics was performed to calculate the mean and standard deviation values for AP translation for each knee flexion angle initially and after 50’000 cycles testing. Wilcoxon Signed-Rank test was used to identify significant differences in AP translation between the two states. The level of significance was set at *p* = 0.05.

## Results

**Fig. 4:**
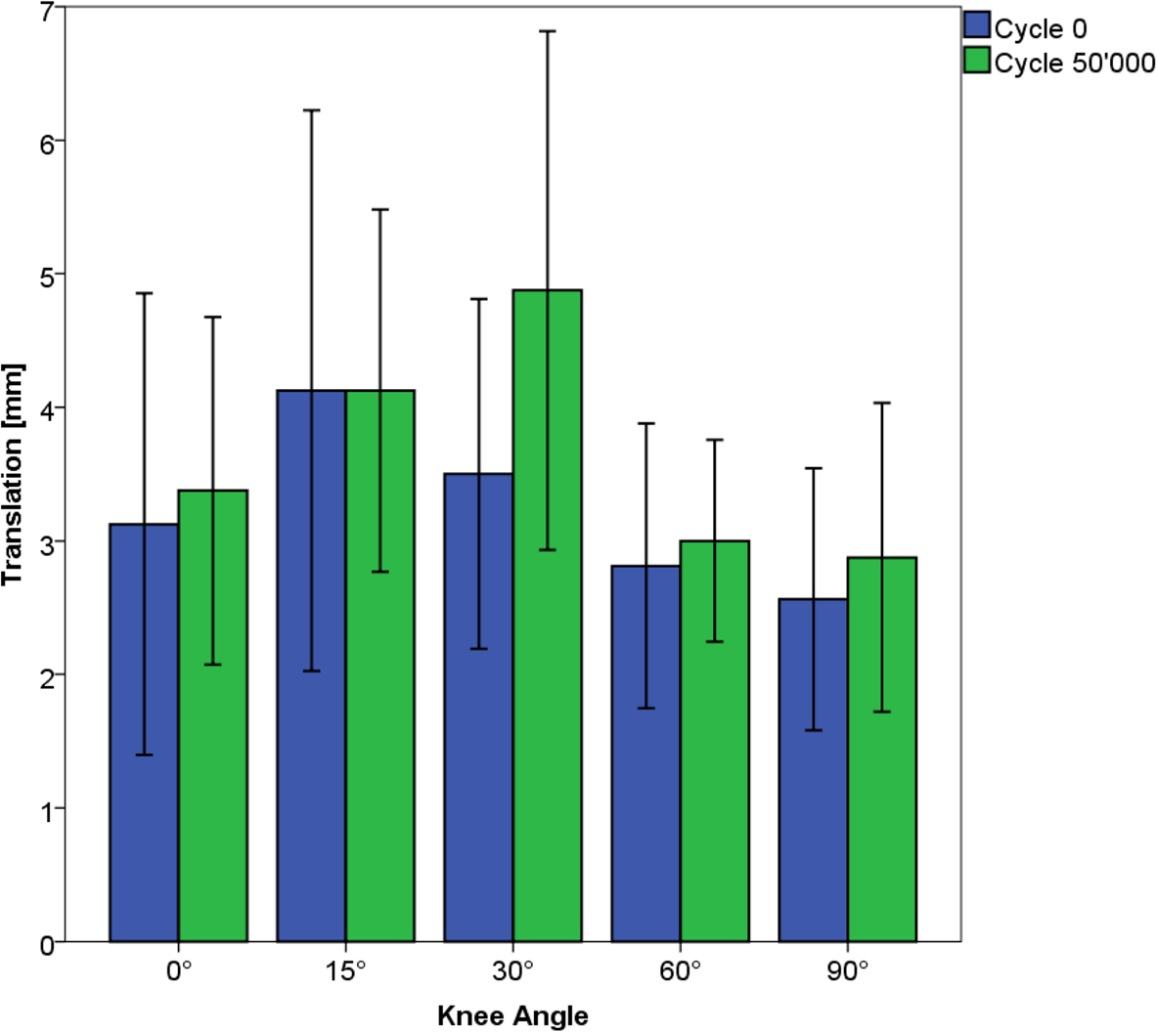
AP translation mean and standard deviation values for 0°, 15°, 30°, 60° and 90° knee flexion before and after 50’000 cycles testing

AP translation before testing amounted to 3.1 ± 1.7 mm in 0°, 4.1 ± 2.1 mm in 15°, 3.5 ± 1.3 mm in 30°, 2.8 ± 1.1 mm in 60° and 2.6 ± 1.0 mm in 90°. After testing, mean AP translation was 3.4 ± 1.3 mm in 0°, 4.1 ± 1.4 mm in 15°, 4.9 ± 1.9 mm in 30°, 3.0 ± 0.8 mm in 60° and 2.9 ± 1.2 mm in 90° (Fig. 4). A significant increase in AP translation of 1.4 mm was measured in 30° (*p* = 0.03), for all other flexion angles the increase in AP translation was below 0.3 mm and not significant (*p* = 0.78 to *p* = 0.89).

## Discussion

The current study biomechanically analyzed AP translational knee kinematics with dynamic augmentation of primary ACL repair in an environment simulating 50’000 gait cycles i.e. the early post-operative phase. AP translation was maintained very close to the immediate post-operative level for all flexion angles except for 30°, where an increase of 1.4 mm was measured. This is the first study assessing the course of AP translational knee laxity with dynamic augmentation of primary ACL repair subjected to dynamic loading.

There are no existing data on in-vivo ACL and ACL-graft forces during rehabilitation. We therefore limited our loading protocol to cyclic flexion-extension of 0°-70°-0° at 1 Hz occurring during walking, and 134 N AP translational force at the time-points and flexion angles of examination (Arnold et al. 2005; Kadaba et al. 1990). The cyclic motion protocol was designed to simulate the early post-operative phase. Preliminary data from a prospective randomized trial at the university hospital in Münster (Germany) showed that DIS patients wearing step counters performed on average 52’000 steps (i.e. 26’000 gait cycles) during the first 3 post-operative weeks (personal notice Dr. Schliemann). Given that physical activity of patients will increase over the period of rehabilitation, the 50’000 cycles, simulated in this study, represent an average post-operative rehabilitation period of 4 to 5 weeks.

The present study design represented a worst-case scenario with regard to load transmission through the implant system over time, considering a relatively high degree of flexion (70°) and exclusion of biological ACL healing. A further reason for the latter assumption was that no data on the mechanical strength of a healing ACL is available in the literature.

Our results best compare to those of Arnold et al., who tested bone-patellar-tendon-bone autografts preconditioned on a tension board for 20 min (Arnold et al. 2005). Knees were tested for 1500 flexion-extension cycles with 0°-70°-0° flexion and at cycles 0, 500, and 1500, knee laxity was measured under 90 N of anterior tibial force at 20° of flexion. Arnold et al. found that anterior laxity increased 1.3 mm after 500 cycles and 1.6 mm after 1500 cycles. Our testing of DIS showed an increase of 0.0 mm in 15° flexion and 1.4 mm in 30° flexion after 50’000 cycles.

Another study performed by Boguszewski et al. used a robotic system to apply 250 cycles of alternating anteroposterior and posteroanterior force of 134 N on ten human knees and then measured the increase in AP tibial translation (Boguszewski et al. 2015). The ACL was reconstructed either with bone-patellar-tendon-bone, bone-achilles-tendon, hamstring-tendon or tibialis tendon and different pretensioning protocols varying in load and duration were conducted. Average increases in AP translation ranged from 1.9 to 3.1 mm depending on the preconditioning and graft type and 75 % of the total increase occurred within the first 125 cycles. All values were thus well above the maximum increase of 1.4 mm we found in our investigation.

It is so not surprising that we recorded the highest values for AP translation in 15° and 30° as this is probably due to the generally reduced additional ligamentous constraint arising from reduced capsular and collateral ligament tension in slight flexion. However, a direct comparison of the dynamic augmentation technique to any type of ACL-reconstruction is associated with limitations because the former serves as an internal brace to protect the healing ACL whereas the latter substitutes the native ACL. AP translational knee laxity with ACL repair techniques finally depends on a stable scar tissue formation at the healing site whereas with ACL reconstruction remodeling of the graft plays a pivotal role (Heitmann et al. 2014; Janssen & Scheffler 2014). Ideally, an augmentation would fully stressshield the ACL repair during the first days after surgery to provide a calm environment for fibrin clot formation and then continuously decrease in stiffness and therefore transfer back the load to the ACL in order to generate a stable scar tissue with longitudinal orientation of collagen and elastin fibres. However, load distribution between the two parallel systems “ACL repair” and “dynamic augmentation” as well as the amount of mechanical stimuli needed for optimal ACL healing are unknown and could be subject to future investigations. Nevertheless, our measurements show that dynamic augmentation is capable of supporting an ACL repair during the initial and very likely most important phase of biological healing.

The limitations of this study ARE similar to those inherent to all cadaveric studies. A limited number of specimens were used, thus restricting generalization to actual patients. In addition, degradation of the knee specimens was a concern, muscle tension was not present and therefore knee motion was uniquely passive and femoral and tibial tunnel positions during implantation were not assessed. However, drying-out of the specimens was prevented by leaving the joint surrounding soft tissue intact, including the skin. Paired knees of four donors were used resulting in a reduced statistical power and overall sample size as left and right knees of the same donor might show similar behavior. The translational knee laxity was measured with a clinically widely used instrument (Rolimeter) and, although the anteriorly directed force executed by the examiner was standardized to 134 N in order to reduce inter-tester variability, variability can still occur (Herbort et al. 2013; Loh et al. 2003; Petersen et al. 2007; Schliemann et al. 2015).

The strength of our study is that it replicated the clinical situation much better than a simple ex-situ uniaxial quasi-static loading test. The donor age of the specimens represented the young age of the typical patient population experiencing ACL ruptures. Bone quality, joint integrity and kinematics therefore highly represented a realistic scenario. Freezing and defrosting of the specimens does not have a relevant effect on the quality and on the mechanical properties of bone and ligament tissue (Linde & Sørensen 1993; Woo et al. 1986). The intra-articular milieu was best replicated with artificial synovial fluid and Lachman/KT-1000 testing was simulated applying a standardized force of 134 N to reduce inter-tester variability. Instrumentation was performed by an experienced knee surgeon (PH).

## Conclusion

With the current study, the early post-operative AP translational knee laxity with dynamic augmentation of primary ACL repair was biomechanically examined. AP translational knee laxity remained close to the immediate post-operative level over a simulated rehabilitation period of 50’000 gait cycles. This technique is therefore capable of supporting the ACL repair during biological healing.

## Abbreviations

ACL: Anterior cruciate ligament
AP: Anteroposterior
BPTB: Bone-patellar-tendon-bone
DIS: Dynamic intraligamentary stabilization

## References

[CR1] Ahlden M, Samuelsson K, Sernert N, Forssblad M, Karlsson J, Kartus J (2012). The Swedish National Anterior Cruciate Ligament Register: a report on baseline variables and outcomes of surgery for almost 18,000 patients. Am J Sport Med.

[CR2] Arnold MP, Lie DT, Verdonschot N, de Graaf R, Amis AA, van Kampen A (2005). The remains of anterior cruciate ligament graft tension after cyclic knee motion. Am J Sport Med.

[CR3] Boguszewski DV, Joshi NB, Wang D, Markolf KL, Petrigliano FA, McAllister DR (2015). Effect of Different Preconditioning Protocols on Anterior Knee Laxity After ACL Reconstruction with Four Commonly Used Grafts. J Bone Joint Surg Am.

[CR4] Brückner H (1966). Eine neue Methode der Kreuzbandplastik. Chirurg.

[CR5] Eggli S, Kohlhof H, Zumstein M, Henle P, Hartel M, Evangelopoulos DS, Bonel H, Kohl S (2015). Dynamic intraligamentary stabilization: novel technique for preserving the ruptured ACL. Knee Surg Sports Traumatol Arthrosc.

[CR6] Engebretsen L, Benum P, Fasting O, Mølster A, Strand T (1990). A prospective, randomized study of three surgical techniques for treatment of acute ruptures of the anterior cruciate ligament. Am J Sport Med.

[CR7] Feagin J, Curl WW (1975). Isolated tear of the anterior cruciate ligament: 5-year follow-up study. Am J Sport Med.

[CR8] Freedman KB, D’Amato MJ, Nedeff DD, Kaz A, Bach BR (2003). Arthroscopic anterior cruciate ligament reconstruction a metaanalysis comparing patellar tendon and hamstring tendon autografts. Am J Sport Med.

[CR9] Grindem H, Eitzen I, Engebretsen L, Snyder-Mackler L, Risberg MA (2014). Nonsurgical or surgical treatment of ACL injuries: knee function, sports participation, and knee reinjury. J Bone Joint Surg.

[CR10] Heitmann M, Dratzidis A, Jagodzinski M, Wohlmuth P, Hurschler C, Puschel K, Giannakos A, Preiss A, Frosch KH (2014). Ligament bracing--augmented cruciate ligament sutures: biomechanical studies of a new treatment concept. Unfallchirurg.

[CR11] Henle P, Roder C, Perler G, Heitkemper S, Eggli S (2015). Dynamic Intraligamentary Stabilization (DIS) for treatment of acute anterior cruciate ligament ruptures: case series experience of the first three years. BMC Musculoskelet Disord.

[CR12] Herbort M, Tecklenburg K, Zantop T, Raschke MJ, Hoser C, Schulze M, Petersen W, Fink C (2013). Single-bundle anterior cruciate ligament reconstruction: a biomechanical cadaveric study of a rectangular quadriceps and bone--patellar tendon--bone graft configuration versus a round hamstring graft. Arthroscopy.

[CR13] Janssen RP, Scheffler SU (2014). Intra-articular remodelling of hamstring tendon grafts after anterior cruciate ligament reconstruction. Knee Surg Sports Traumatol Arthrosc.

[CR14] Kadaba MP, Ramakrishnan H, Wootten M (1990). Measurement of lower extremity kinematics during level walking. J Orthop Res.

[CR15] Kessler MA, Behrend H, Henz S, Stutz G, Rukavina A, Kuster MS (2008). Function, osteoarthritis and activity after ACL-rupture: 11 years follow-up results of conservative versus reconstructive treatment. Knee Surg Sports Traumatol Arthrosc.

[CR16] Kohl S, Evangelopoulos DS, Kohlhof H, Hartel M, Bonel H, Henle P, von Rechenberg B, Eggli S (2013). Anterior crucial ligament rupture: self-healing through dynamic intraligamentary stabilization technique. Knee Surg Sports Traumatol Arthrosc.

[CR17] Kohl S, Evangelopoulos DS, Ahmad SS, Kohlhof H, Herrmann G, Bonel H, Eggli S (2014). A novel technique, dynamic intraligamentary stabilization creates optimal conditions for primary ACL healing: a preliminary biomechanical study. Knee.

[CR18] Kohl S, Stock A, Ahmad SS, Zumstein M, Keel M, Exadaktylos A, Kohlhof H, Eggli S, Evangelopoulos DS (2015). Dynamic intraligamentary stabilization and primary repair: A new concept for the treatment of knee dislocation. Injury.

[CR19] Kohn D, Wirth C-J, Adam F (2005) Orthopädie und orthopädische Chirurgie: das Standardwerk für Klinik und Praxis. Knie: 67 Tabellen. Thieme,

[CR20] Kosters C, Herbort M, Schliemann B, Raschke MJ, Lenschow S (2015). Dynamic intraligamentary stabilization of the anterior cruciate ligament : Operative technique and short-term clinical results. Unfallchirurg.

[CR21] Laxdal G, Kartus J, Ejerhed L, Sernert N, Magnusson L, Faxen E, Karlsson J (2005). Outcome and risk factors after anterior cruciate ligament reconstruction: a follow-up study of 948 patients. Arthroscopy.

[CR22] Legnani C, Ventura A, Terzaghi C, Borgo E, Albisetti W (2010). Anterior cruciate ligament reconstruction with synthetic grafts. A review of literature. Int Orthop.

[CR23] Linde F, Sørensen HCF (1993). The effect of different storage methods on the mechanical properties of trabecular bone. J Biomech.

[CR24] Loh JC, Fukuda Y, Tsuda E, Steadman RJ, Fu FH, Woo SL (2003). Knee stability and graft function following anterior cruciate ligament reconstruction: Comparison between 11 o’clock and 10 o’clock femoral tunnel placement. 2002 Richard O’Connor Award paper. Arthroscopy.

[CR25] Marshall JL, WARREN RF, WICKIEWICZ TL, REIDER B (1979). The anterior cruciate ligament: a technique of repair and reconstruction. Clin Orthop Relat Res.

[CR26] Marshall JL, Warren RF, Wickiewicz TL (1982). Primary surgical treatment of anterior cruciate ligament lesions. Am J Sport Med.

[CR27] Meuffels DE, Favejee M, Vissers M, Heijboer M, Reijman M, Verhaar J (2009). Ten year follow-up study comparing conservative versus operative treatment of anterior cruciate ligament ruptures. A matched-pair analysis of high level athletes. Br J Sports Med.

[CR28] Murray MM, Spindler KP, Devin C, Snyder BS, Muller J, Takahashi M, Ballard P, Nanney LB, Zurakowski D (2006). Use of a collagen-platelet rich plasma scaffold to stimulate healing of a central defect in the canine ACL. J Orthop Res.

[CR29] Murray MM, Spindler KP, Ballard P, Welch TP, Zurakowski D, Nanney LB (2007). Enhanced histologic repair in a central wound in the anterior cruciate ligament with a collagen-platelet-rich plasma scaffold. J Orthop Res.

[CR30] Petersen W, Tretow H, Weimann A, Herbort M, Fu FH, Raschke M, Zantop T (2007). Biomechanical evaluation of two techniques for double-bundle anterior cruciate ligament reconstruction: one tibial tunnel versus two tibial tunnels. Am J Sport Med.

[CR31] Petrigliano FA, McAllister DR, Wu BM (2006). Tissue engineering for anterior cruciate ligament reconstruction: a review of current strategies. Arthroscopy.

[CR32] Pinczewski LA, Lyman J, Salmon LJ, Russell VJ, Roe J, Linklater J (2007). A 10-year comparison of anterior cruciate ligament reconstructions with hamstring tendon and patellar tendon autograft: a controlled, prospective trial. Am J Sports Med.

[CR33] Schliemann B, Lenschow S, Domnick C, Herbort M, Haberli J, Schulze M, Wahnert D, Raschke MJ, Kosters C (2015). Knee joint kinematics after dynamic intraligamentary stabilization: cadaveric study on a novel anterior cruciate ligament repair technique. Knee Surg Sports Traumatol Arthrosc.

[CR34] Silva A, Sampaio R (2009). Anatomic ACL reconstruction: does the platelet-rich plasma accelerate tendon healing?. Knee Surg Sports Traumatol Arthrosc.

[CR35] Steadman JR, Cameron-Donaldson ML, Briggs KK, Rodkey WG (2006). A minimally invasive technique (“healing response”) to treat proximal ACL injuries in skeletally immature athletes. J Knee Surg.

[CR36] Steadman JR, Matheny LM, Briggs KK, Rodkey WG, Carreira DS (2012). Outcomes following healing response in older, active patients: a primary anterior cruciate ligament repair technique. J Knee Surg.

[CR37] Struewer J, Frangen TM, Ishaque B, Bliemel C, Efe T, Ruchholtz S, Ziring E (2012). Knee function and prevalence of osteoarthritis after isolated anterior cruciate ligament reconstruction using bone-patellar tendon-bone graft: long-term follow-up. Int Orthop.

[CR38] Vavken P, Murray MM (2011). The potential for primary repair of the ACL. Sports Med Arthrosc.

[CR39] West RV, Harner CD (2005). Graft selection in anterior cruciate ligament reconstruction. J Am Acad Orthop Sur.

[CR40] Woo SL-Y, Orlando CA, Camp JF, Akeson WH (1986). Effects of postmortem storage by freezing on ligament tensile behavior. J Biomech.

